# Time-restricted feeding and the realignment of biological rhythms: translational opportunities and challenges

**DOI:** 10.1186/1479-5876-12-79

**Published:** 2014-03-28

**Authors:** Jag Sunderram, Stavroula Sofou, Kubra Kamisoglu, Vassiliki Karantza, Ioannis P Androulakis

**Affiliations:** 1 Department of Medicine, Division of Pulmonary and Critical Care Medicine, Rutgers - Robert Wood Johnson Medical School, New Brunswick, NJ 08901, USA; 2 Biomedical Engineering Department, Rutgers University, Piscataway, NJ 08854, USA; 3 Chemical & Biochemical Engineering Department, Rutgers University, Piscataway, NJ 08854, USA; 4 Rutgers Cancer Institute of New Jersey, Rutgers University, New Brunswick 08901, USA

## Abstract

It has been argued that circadian dysregulation is not only a critical inducer and promoter of adverse health effects, exacerbating symptom burden, but also hampers recovery. Therefore understanding the health-promoting roles of regulating (i.e., restoring) circadian rhythms, thus suppressing harmful effects of circadian dysregulation, would likely improve treatment. At a critical care setting it has been argued that studies are warranted to determine whether there is any use in restoring circadian rhythms in critically ill patients, what therapeutic goals should be targeted, and how these could be achieved. Particularly interesting are interventional approaches aiming at optimizing the time of feeding in relation to individualized day–night cycles for patients receiving enteral nutrition, in an attempt to re-establish circadian patterns of molecular expression. In this short review we wish to explore the idea of transiently imposing (appropriate, but yet to be determined) circadian rhythmicity via regulation of food intake as a means of exploring rhythm-setting properties of metabolic cues in the context of improving immune response. We highlight some of the key elements associated with his complex question particularly as they relate to: a) stress and rhythmic variability; and b) metabolic entrainment of peripheral tissues as a possible intervention strategy through time-restricted feeding. Finally, we discuss the challenges and opportunities for translating these ideas to the bedside.

## Introduction

**Figure 1:**
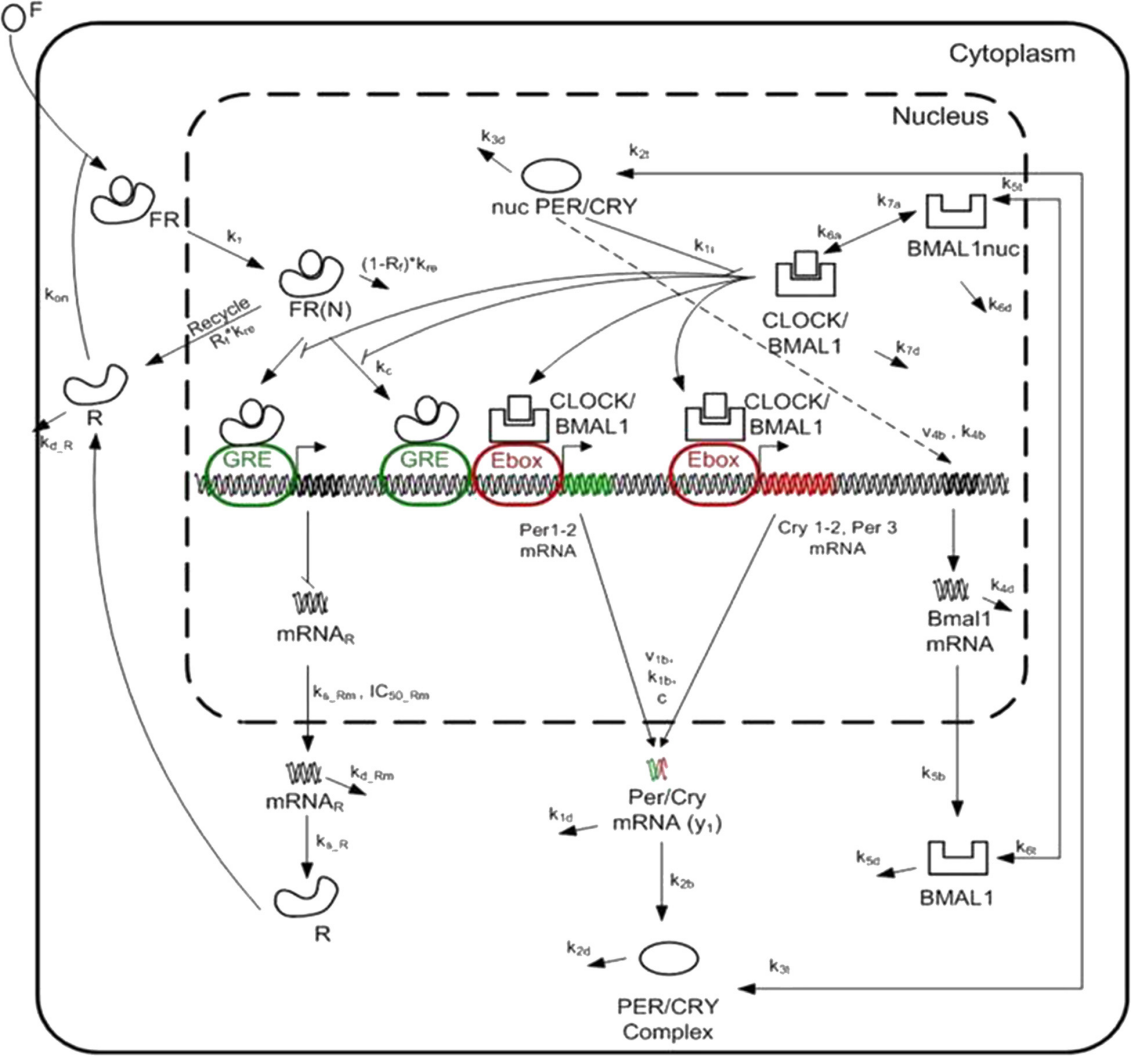
**The periodic expression of clock genes is driven by *Per *and *Cry *inhibiting the activity of the CLOCK/BMAL1 dimer (negative feedback) and stimulating Bmal1 gene transcription (positive feedback).** Through a negative feedback loop, the heterocomplex CLOCK/BMAL1 activates the transcription of period (*Per*) and cryptochrome (Cry) genes upon binding to the E-box promoter region. After the expression of PER/CRY proteins in the cytoplasm, they translocate to the nucleus where they inhibit their own transcription by shutting off the transcriptional activity of the CLOCK/BMAL1 heterocomplex . Through the positive feedback loop the nuclear compartment of PER/CRY protein (y3) activates indirectly *Bmal1* mRNA (y4) transcription, which after its translation to BMAL1 protein and its translocation to the nucleus, increases the expression of CLOCK/BMAL1 heterodimer. However, the peripheral clocks are “entrained” by external signals – cortisol (F) in this case. The role of the entertainer is to synchronize the responses across a collection of cells. Figure adapted from [23].

**Figure 2:**
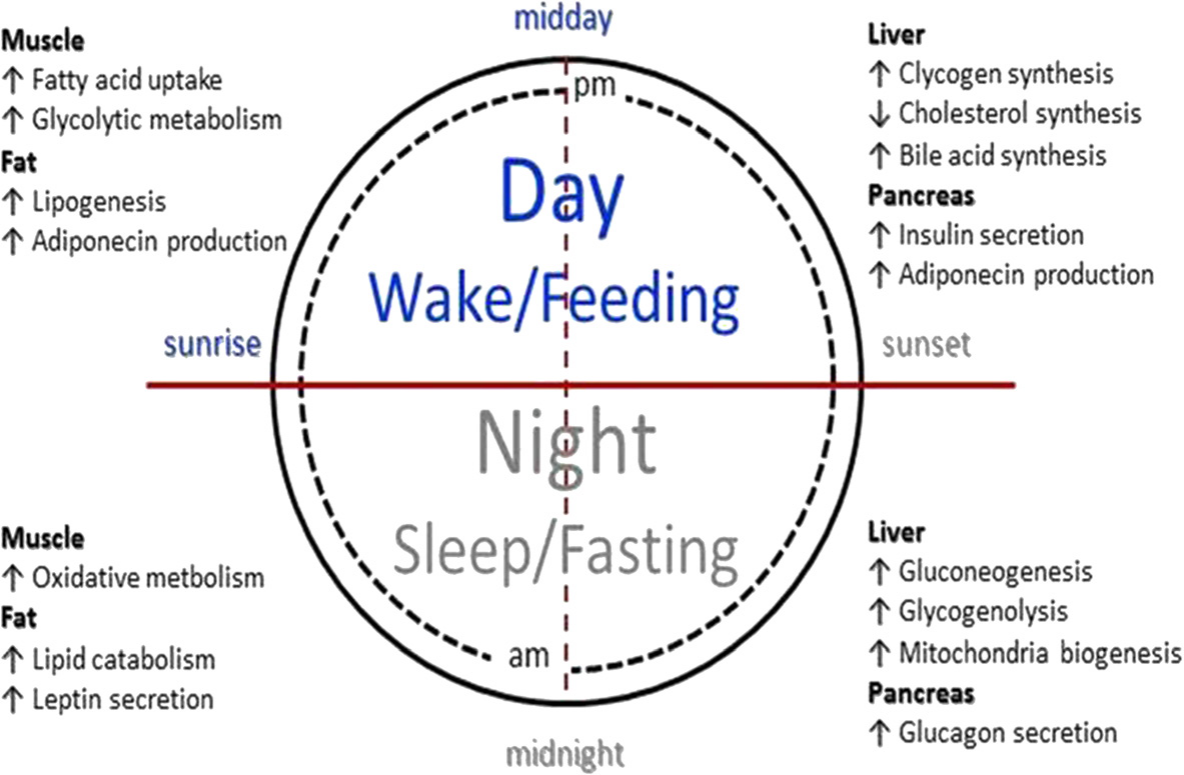
**Biological clocks enable the integration of behavioural cycles (sleep/wake – feed/fast) and metabolic processes across different organs.** While each tissue maintains its own clock ad intrinsic rhythms, external signals (*zeitgebers*) coordinate functions inorder to maintain appropriate balances. (Figure adapted from [24]).

Biological rhythms are major determinants of behavioural outcome [1,2] and are controlled by a tightly regulated network of genes and proteins entrained by external signals (light and food). The suprachiasmatic nucleus (SCN) is the fundamental, central, regulator of circadian rhythmicity (biological rhythms of, roughly, 24 h period) and is considered the master clock designed to align, and coordinate the independent, self-sustained, peripheral oscillators (a.k.a. peripheral clocks) found in every cell, tissue and organ [3-6]. In that respect, understanding the mechanisms by which the various pacemakers interact to coordinate functions becomes a critical question [7]. Despite the fact that all peripheral clocks effectively utilize the same time-keeping machinery [8-11] (Figure 1) each peripheral entity is impacted by unique stimuli capable of setting clock rhythmicity locally, directly or indirectly. As such, core physiological functions are strongly impacted by the appropriate alignment of peripheral clocks to central (SCN) rhythms [12,13] likely mediated via circulating hormones [14,15]. While biological rhythms convey anticipatory signals priming the host for periods of food intake, increased activity and rest [16-18] (Figure 2) the loss of these rhythms has deleterious effects on overall health [19]. The interplay between a host’s well-being and its biological rhythms is critical and bi-directional: disrupted rhythms impact the response to stress whereas stress alters the characteristics of biological rhythms [20-22].

**Figure 3:**
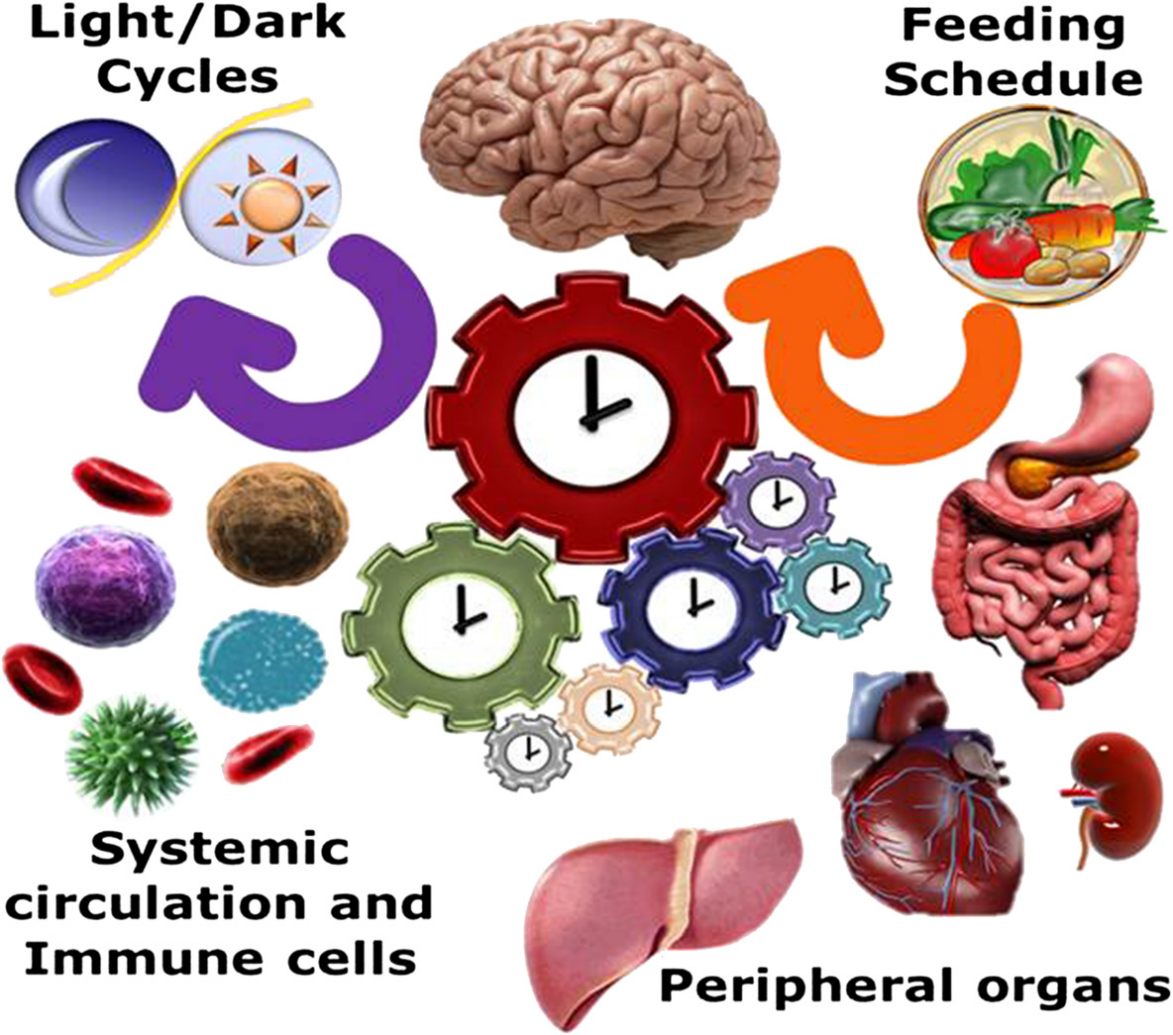
**Systemic signals act as *coordinators *** **of peripheral oscillators maintaining synchrony of function and health.**

Emerging evidence suggesting that rhythmic signals play a major role in immune [25-27] and metabolic [28] functions naturally leads to the possibility of exploring biological rhythms as targets of intervention strategies, and in particular in the context of intensive care units (ICU) where non-natural light schedules and time-invariant nutritional and/or pharmaceutical interventions may deprive patients of the rhythmic cues necessary to maintain appropriate biological rhythmicity during the recovery phase [29,30] and loss of entraining inputs may significantly impact recovery [10,31-34]. In fact circadian abnormalities correlate with severity of illness and outcome [35]. Due to the strong role rhythmicity plays in recovering from trauma [36,37], its regulation and realignment are emerging as potentially critical controllers influencing patient outcome by regulating entraining signals in a non-invasive manner. Circadian cues that control rest cycles and metabolism are primarily driven by light and food [38,39]. These play a fundamental role in that they maintain proper synchrony between the peripheral clocks (Figure 3). The importance of maintaining good coordination between the peripheral oscillators is so critical that physicians have speculated that “[…] *healthy organs behave as biological oscillators, which couple to one another during human development, and that this orderly coupling is maintained through a communications network, including neural, humoral, and cytokine components.* [We] *suggest that the systemic inflammatory response syndrome initiates disruption of communication and uncoupling, and further suggest that progression into the multiple organ dysfunction syndrome reflects progressive uncoupling that can become irreversible. Resolution of the inflammatory response and reestablishment of the communications network are necessary but may not be, by itself, sufficient to allow organs to appropriately recouple*” [40].

Exploring these cycles in order to realign patients’ biological rhythms during the recovery phase may prove to be highly rewarding in terms of outcome [41]. Therefore, understanding the mechanisms that entrain the central and peripheral clocks, and the ways in which these rhythms influence the ability of the host organism to respond to, and recover from, external threats and challenges is critical to developing new models of patient care capable of engaging these rhythms in an attempt to, potentially, improve outcome. It must be noted that although it is well established that ICU patients have abnormal circadian patterns [35,42] the overall environment in the ICU, including the patient’s condition, the lighting and noise levels in ICU as well as – and likely very importantly – the treatment the patient receives, induces significant circadian alterations [43,44].

In this short review we focus on one particular approach to resetting biological rhythmicity in the context of *time-restricted feeding* (TRF; access to food is restricted for specific time intervals during the day without calorie restrictions) and explore the possibility of pursuing circadian re-alignment via nutritionally-inspired interventions. Although the focus of the review is on the implications of restoring circadian rhythms we should point out that appropriate sampling and analysis of biochemical and physiological circadian data requires careful design and execution and these have been the subject of numerous excellent reviews [45].

## Circadian reprogramming as an intervention strategy: opportunities for time-restricted feeding

### Stress-induced loss of circadian rhythmicity

Evidence establishing the strong links between biological rhythms and stress response is overwhelming and, by now, very well established and accepted [14,46]. However, the translational implications, opportunities and challenges of how to manipulate rhythms in an ICU environment are only now beginning to emerge in the scientific discourse [30]. A number of recent, and older, reviews have discussed the connections between immune function and biological rhythms [46] where the bi-directional relationship between *disrupted rhythms* and *immune dysfunction;* and its implication on the bedside have been clearly identified [36,37]. What is even more interesting is the fact that we begin to realize that circadian dysfunction following stress may have long lasting ramifications [47] pointing to possible sources of comorbidities. In fact, it has been argued that different procedures impact post-operative circadian disruption in a differential manner, thus affecting recovery, raising the possibility of guiding operative procedures based on their capacity to minimize impact on biological rhythms [48]. Clinical studies specifically emphasized that biological night and day cycles (measured by urinary 6-sulfatoxymelatonin) were phase-delayed and normal features of sleep were lacking (REM sleep was identified only in 2 patients out of 21) in the critical care patients [49]. Studies on patients undergoing elective maxillofacial surgery showed that strengthening circadian rhythms in anticipation of disruption following surgery can be efficacious for improving the recovery phase. Patients whose circadian rhythms were adjusted pre-operatively by combined sleep/wake cycle alteration and timed food and caffeine ingestion had reduced disruption in their body temperature cycles throughout their recovery in comparison to the control group [50].

One of the most active areas of research pointing directly to circadian disruption and biological rhythm-setting interventions relate to mood disorders [51-55]. A vast literature exists on enhancing circadian rhythms for treating depression, bipolar disorder and other related mood disorders either via pharmacological (melatonin) [55,56] or non-pharmacological means (light) [57] aimed at boosting circadian rhythms.

### Circadian (re)alignment and time-restricted feeding

Time restricted feeding (TRF) is essentially imposing rhythms on nutrient availability. Entrainment by TRF has generated significant interest due to the possibility of synchronizing peripheral clocks without clear influences on (or from) the central pacemaker (SCN) [28,58]. It has been speculated that restricted feeding (RF) entrains rhythms in peripheral tissues (liver and lung) [6] is likely independent of the SCN. These works challenge the basic hierarchical paradigm that light entrains the SCN which subsequently entrains the peripheral clocks and emphasized the role of RF as an entraining signal. The hypothesis of independently entrained peripheral clocks has been further reinforced by the observation that even lesions in brain nuclei do not eliminate food anticipatory activity, thus pointing to likelihood of a distributed system maintaining and regulating food-anticipatory activities [59,60]. One of the main justifications is that when food accessibility adopts specific rhythmic characteristics so will the physiology and behaviour to match nutritional resource availability [61]. It has been shown that feeding mice during the day completely reverses the phase of circadian oscillators (specifically, four clock components, *Per1, Per2, Per3, Cry1*; and the two circadian transcription factors DBP and Rev-erbα) in multiple peripheral cells (liver, kidney, heart and pancreas), but has little if any effect on the central oscillator in the SCN [62]. However, we must point out that RF entrains the rhythm of clock protein *Per2* even in the SCN as was shown in studies that eliminated photic stimulation by keeping mice in constant darkness [63], or at constant light conditions [64], thus raising the possibility of peripheral oscillators resetting the central clock.

In a carefully designed study of a murine obesity model [53] the authors convincingly show the intimate relationship between the signalling and transcriptional components of energy metabolism and the circadian system. The study hypothesized that TRF improves diurnal rhythms; drives lipid homeostasis while preventing weight gain, hepatosteatosis and liver damage; improves adipose homeostasis and reduces inflammation. The study demonstrated that preserving natural feeding rhythms significantly dampens metabolic disruption induced by a high fat diet, including improving oscillations of the liver circadian clock components. Therefore, while the total calorie intake and food composition (high fat) remained constant, the study clearly demonstrated that an apparent *lifestyle*, i.e.,non-pharmacological, intervention prevented obesity, and related co-morbidities, possibly by resetting metabolic cycles. The role of *food-anticipatory* activity has also been explored with a focus on energy metabolism, defined by oxygen consumption [65]. Animals were allowed access to food for only few hours during either the light or the dark phases. Locomotor activity, body temperature, clock gene expression in liver and energy metabolism were recorded and their changes assessed as the time window over which food became available was changing. Continuous monitoring of energy metabolism and core body temperature indicated expected, robust diurnal rhythmic characteristics but also rapid re-entrainment and adaptation to restricted food access.

A series of publications has focused on comparing protein synthesis under a continuous and, a likely more physiologically realistic, intermittent bolus feeding regimen, delivered by orogastric tube, in neonatal pigs in the context of regulating protein synthesis [66-68]. The analysis demonstrated that intermittent feeding (delivered every 4 hrs as a bolus feed) enhances muscle protein synthesis by imposing pulsatile patterns of amino-acid and insulin-induced translation initiation. In this very interesting series of papers it has been argued that bolus feeding promotes a more physiological surge of intestinal hormones. The studies effectively hypothesize that “[…] *cyclic surge of amino acids and insulin is needed to maximally stimulate protein synthesis in skeletal muscle*” and that “[…] *bolus compared to continuous feeding has been advocated to promote more normal feed-fast hormonal profiles*”. It has been further demonstrated that either advancing or delaying meal time in young adult mice results in reversible alterations of temperature and overall cage activities [69]. Longer time restriction (one week) alters rhythms in glucose, triglyceride and HDL levels. Food restriction results in behavioral arousal in anticipation of food presentation and induces a shift in the circadian phase of many physiological variables, likely independent of the SCN. As such, RF is expected to exert changes in organs “handling nutrients” (such as liver). As previous work had suggested RF could be associated with significant stress due to hyperphagia, In a study examining the effect of restricted feeding on stress markers, no marked changes in body weight, retroperitoneal decrease in lipid deposits and peak in glucocorticoids accompanying expectation to food access were identified [70]. Given the probable relationship between stress and metabolic alterations (in this case interest was in liver) the study explored whether an increase in acute phase proteins (APR) or pro-inflammatory state occurred after 2 weeks of 2hr food restriction. The “positive control” for APR consisting of a group injected with LPS showed a significant increase in systems APR while neither the ad libitum nor restricted feeding induced a marked increase in any of the inflammatory markers. Furthermore, a marked change in the diurnal patterns of circulating cytokines was observed as a consequence of RF. The authors advance an interesting hypothesis stating that RF may establish a distinctive state (“rheostatic response” earlier introduced in [71]) likely enabling the system to adopt a transient functional state “change in set-point”, boosting the rhythms and the overall fitness of the host.

### Time-restricted feeding and disease progression

Peripheral circadian de-synchrony may be an early indicator of metabolic disruption in shift workers due to sleep deprivation mediated disruption of circadian rhythms. By extension, strengthening the peripheral circadian rhythm, by imposing metabolic rhythms via limiting food intake during the night, may counteract comorbidities seen in human shift workers [72]. This study further implies that the manipulation of circadian rhythms need not be such that it aims at restoring the homeostatic nature of the internal clock. Rather it implies that, at least in the short term, strengthening other rhythmic frequencies may be more beneficial.

Particularly interesting is the work investigating the effect of resetting circadian clocks in peripheral tissues using non-photic signals on tumor growth rate in rats [73,74]. Restricting the timing of meals to light time in contrast to restricted feeding during the night (active phase of rats) thereby, imposing a reversed metabolic rhythm, induced, what is referred to as, “internal desynchronization” (described as loss of phase relationship between central – light entrained – and peripheral clocks) resulted in prolonged survival and slowed down tumor growth. The authors speculate that meal timing during the light period amplifies host rhythms and assigns their peak in a time window when the tumor is most susceptible to host-mediated control and that tumor growth is hampered when the internal (metabolic) clock adopts specific rhythmic characteristics, interestingly the opposite of what would have been otherwise considered “natural”. Therefore, the emerging hypothesis is that, a radically different metabolic rhythmicity appears to be most effective at least in the short term.

### Restricted-time feeding vs. calorie restriction

It is important to draw a distinction between time-restricted feeding and caloric restriction. The former entails the delivery of a certain amount of calories albeit at specific time intervals of specific duration. Therefore subjects still receive a standard nutritional intake. Calorie restriction entails an overall reduction in caloric intake, albeit without malnutrition. While evidence for the benefits of calorie restriction in animals has been promising, the issue as it relates to humans is still debated as conducting long term studies assessing the implications of prolonged calorie restriction in a controlled manner is rather complicated [75]. Although studies have shown calorie restriction improves post-trauma outcomes [76-78], it is likely that the long term effects of calorie restriction are related to alterations of biological mechanisms responsible for maintenance of health [79]. Recent work has indicated the possibility of caloric restriction impacting circadian clocks as well [80]. However, it is argued that this may be a secondary effect of calorie restriction resulting in time restricted feeding imposing specific rhythms on metabolic function and entraining peripheral clocks. Nevertheless, the focus of this discussion is on TRF and not on calorie restriction.

### Clinical studies comparing continuous vs. bolus feeding

A number of fairly comprehensive clinical studies have considered the impact of temporal delivery of enteral feeding in critical patients [81-88]. Although these studies have to be acknowledged in the context of our discussion, one should be aware of the fact that clinical studies comparing continuous vs. bolus feeding were motivated mostly by the need to address some of the key practical limitations associated with delivering nutritional support, such as interruptions of continuous feeding leading to an inability to achieve nutritional goals, gastrointestinal complications, modulation of aspiration pneumonia, stool frequency etc., rather than as an attempt to capitalize on potentially advantageous physiological and/or biochemical routes linking metabolic rhythms and immune response. Earlier studies examined various parameters influenced by delivering enteral nutrition in the form of either continuous or bolus (intermittent) delivery and the conclusions are still debated in the clinical community [84]. Studies comparing continuous to intermittent tube feeding in adult burn patients concluded that patients continuously fed had reduced stool frequency and time required to achieve nutritional goals. More recent studies, however, despite minor differences in specific goals and targets, in general do not provide evidence of significant difference in terms of patient outcome. Results show that patients intermittently fed have a higher total intake volume, are extubated earlier, and have a lower risk of aspiration pneumonia. Postoperatively, feeding at night only is more energy efficient than is feeding continuously for 24 h, but is associated with poorer nitrogen balance [82]. In one of the very few studies which complemented intermittent feeding in a clinical setting with monitoring of biomarkers, the observed decrease in urinary catecholamine secretion indicated a possible role of sympatho-adrenal mechanisms. This study provides a link between feeding patterns and putatively modulated pathways. However, no studies have been performed where time restricted feeding has been compared to either bolus or continuous feeding in the ICU.

## Concluding remarks

**Figure 4:**
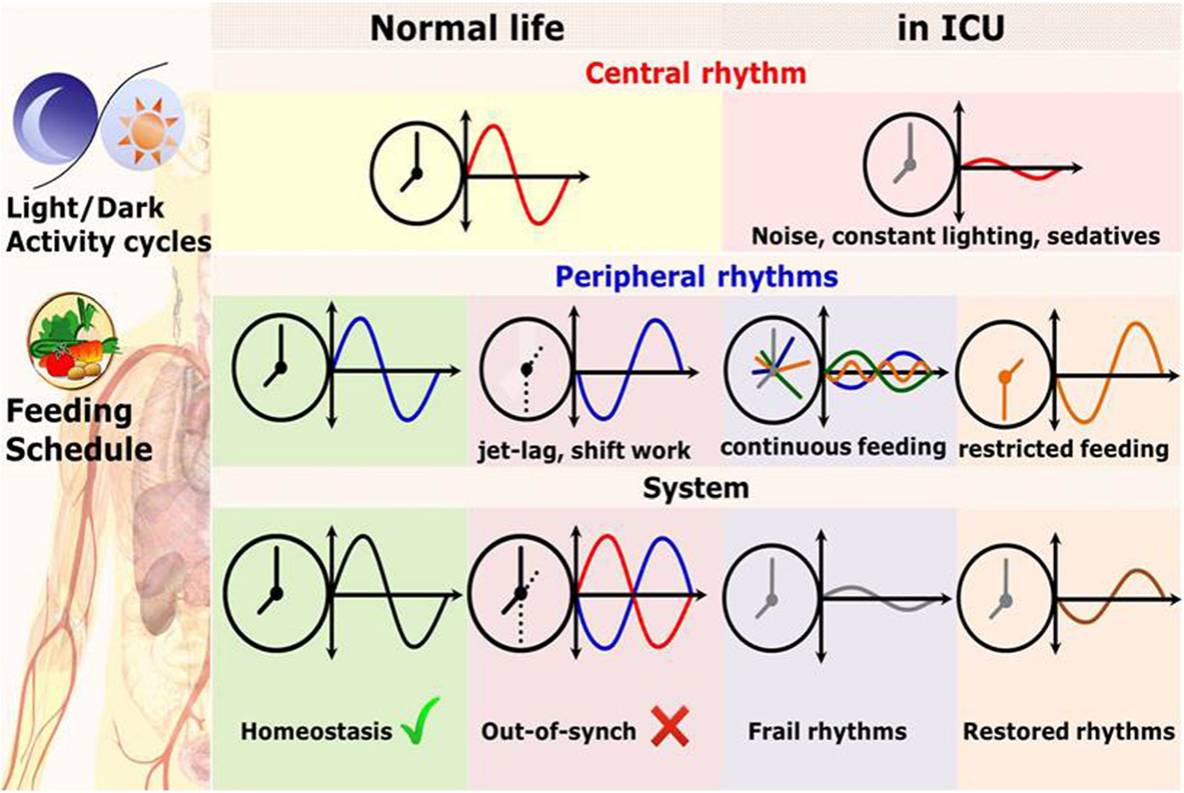
**Robust circadian entrainment characterizes homeostasis, whereas disruptions (amplitude and phase) characterize stressful critical conditions.** Whereas in homeostasis circadian rhythms are properly aligned with clearly identifiable phase locking, circadian disruption shifts peripheral clock rhythms. Restoration of peripheral rhythms, including nutritionally-driven metabolic rhythms through time-restricted feeding will re-entrain the system and provide appropriate systemic cues.

Time restricted delivery of metabolites imposes rhythmic availability of nutrients which resets peripheral clocks in a way that potentially exerts a positive impact on the immune response. Recent clinical evidence indicates that restoring circadian rhythms in critically ill patients is important. We hypothesize that providing circadian cues in the ICU could be explored as a mechanism to improve ICU outcome by reinforcing appropriate rhythms of hormonal release [30,34] (Figure 4). As it is becoming increasingly more evident that exploring alternatives measures to re-establish circadian patters of molecular expression via non-pharmacological means could hold significant potential, feeding entrainment through the possibility of optimizing the time of feeding in relation to the light/dark cycle for patients receiving enteral nutrition appears to beg for more investigation [29].

In this brief review we elaborated on the idea that establishing abolished rhythms would have a beneficial effect on the host response to stress. We highlighted some of the key elements associated with this complex question particularly as they relate to: a) stress and rhythmic variability; and b) metabolic entrainment of peripheral tissues as a possible intervention strategy through time-restricted feeding. Positive effects have been shown in the context of psychological stress, mood disorders etc., using either pharmacologic agents, aiming at restoring circadian signals, or using photic signals to activate the central pace maker. The question, however, remains whether imposing appropriate metabolic rhythms, likely not maintaining homeostatic phase relations with the central clock, through time-restricted feeding would lead to beneficial entrainment of peripheral clocks resulting in improved health outcomes with a host under stress.

## Competing interests

The authors declare that they have no competing interests.

## Authors’ contributions

JS, SS, KK, VK edited the manuscript; IPA conceived the idea, prepared and edited the manuscript. All authors read and approved the final manuscript.

## References

[B1] Reppert SM Weaver DR Coordination of circadian timing in mammals Nature 2002 418 935 941 10.1038/nature00965 12198538

[B2] Lowrey PL Takahashi JS Mammalian circadian biology: elucidating genome-wide levels of temporal organization Annu Rev Genomics Hum Genet 2004 5 407 441 10.1146/annurev.genom.5.061903.175925 15485355 PMC3770722

[B3] Liu C Weaver DR Strogatz SH Reppert SM Cellular construction of a circadian clock: period determination in the suprachiasmatic nuclei Cell 1997 91 855 860 10.1016/S0092-8674(00)80473-0 9413994

[B4] Kohsaka A Bass J A sense of time: how molecular clocks organize metabolism Trends Endocrinol Metab 2007 18 4 11 10.1016/j.tem.2006.11.005 17140805

[B5] Grechez-Cassiau A Rayet B Guillaumond F Teboul M Delaunay F The circadian clock component BMAL1 is a critical regulator of p21WAF1/CIP1 expression and hepatocyte proliferation J Biol Chem 2008 283 4535 4542 10.1074/jbc.M705576200 18086663

[B6] Stokkan K-A Yamazaki S Tei H Sakaki Y Menaker M Entrainment of the circadian clock in the liver by feeding Science 2001 291 490 493 10.1126/science.291.5503.490 11161204

[B7] Schibler U Sassone-Corsi P A web of circadian pacemakers Cell 2002 111 919 922 10.1016/S0092-8674(02)01225-4 12507418

[B8] Eisele L Prinz R Klein-Hitpass L Nuckel H Lowinski K Thomale J Moeller LC Duhrsen U Durig J Combined PER2 and CRY1 expression predicts outcome in chronic lymphocytic leukemia Eur J Haematol 2009 83 320 327 10.1111/j.1600-0609.2009.01287.x 19500131

[B9] Everson CA Functional consequences of sustained sleep deprivation in the rat Behav Brain Res 1995 69 43 54 10.1016/0166-4328(95)00009-I 7546317

[B10] Fu L Pelicano H Liu J Huang P Lee C The circadian gene Period2 plays an important role in tumor suppression and DNA damage response in vivo Cell 2002 111 41 50 10.1016/S0092-8674(02)00961-3 12372299

[B11] Gekakis N Staknis D Nguyen HB Davis FC Wilsbacher LD King DP Takahashi JS Weitz CJ Role of the CLOCK protein in the mammalian circadian mechanism Science 1998 280 1564 1569 10.1126/science.280.5369.1564 9616112

[B12] Green CB Takahashi JS Bass J The meter of metabolism Cell 2008 134 728 742 10.1016/j.cell.2008.08.022 18775307 PMC3760165

[B13] Lamia KA Storch KF Weitz CJ Physiological significance of a peripheral tissue circadian clock Proc Natl Acad Sci U S A 2008 105 15172 15177 10.1073/pnas.0806717105 18779586 PMC2532700

[B14] Arjona A Silver AC Walker WE Fikrig E Immunity's fourth dimension: approaching the circadian-immune connection Trends Immunol 2012 33 607 612 10.1016/j.it.2012.08.007 23000010 PMC3712756

[B15] Le Minh N Damiola F Tronche F Schutz G Schibler U Glucocorticoid hormones inhibit food-induced phase-shifting of peripheral circadian oscillators EMBO J 2001 20 7128 7136 10.1093/emboj/20.24.7128 11742989 PMC125339

[B16] Koyanagi S Ohdo S Alteration of intrinsic biological rhythms during interferon treatment and its possible mechanism Mol Pharmacol 2002 62 1393 1399 10.1124/mol.62.6.1393 12435807

[B17] Vitalini MW de Paula RM Park WD Bell-Pedersen D The rhythms of life: circadian output pathways in Neurospora J Biol Rhythms 2006 21 432 444 10.1177/0748730406294396 17107934

[B18] Edery I Circadian rhythms in a nutshell Physiol Genomics 2000 3 59 74 11015601 10.1152/physiolgenomics.2000.3.2.59

[B19] Rana S Mahmood S Circadian rhythm and its role in malignancy J Circadian Rhythms 2010 8 3 10.1186/1740-3391-8-3 20353609 PMC2853504

[B20] Rechtschaffen A Bergmann BM Gilliland MA Bauer K Effects of method, duration, and sleep stage on rebounds from sleep deprivation in the rat Sleep 1999 22 11 31 9989363 10.1093/sleep/22.1.11

[B21] Reilly DF Westgate EJ FitzGerald GA Peripheral circadian clocks in the vasculature Arterioscler Thromb Vasc Biol 2007 27 1694 1705 10.1161/ATVBAHA.107.144923 17541024

[B22] Haimovich B Calvano J Haimovich AD Calvano SE Coyle SM Lowry SF In vivo endotoxin synchronizes and suppresses clock gene expression in human peripheral blood leukocytes Crit Care Med 2010 38 751 758 10.1097/CCM.0b013e3181cd131c 20081528 PMC2929957

[B23] Mavroudis PD Scheff JD Calvano SE Lowry SF Androulakis IP Entrainment of peripheral clock genes by cortisol Physiol Genomics 2012 44 607 621 10.1152/physiolgenomics.00001.2012 22510707 PMC3426436

[B24] Bass J Takahashi JS Circadian integration of metabolism and energetics Science 2010 330 1349 1354 10.1126/science.1195027 21127246 PMC3756146

[B25] Lee JE Edery I Circadian regulation in the ability of Drosophila to combat pathogenic infections Curr Biol 2008 18 195 199 10.1016/j.cub.2007.12.054 18261909 PMC2279094

[B26] Paladino N Leone MJ Plano SA Golombek DA Paying the circadian toll: the circadian response to LPS injection is dependent on the Toll-like receptor 4 J Neuroimmunol 2010 225 62 67 10.1016/j.jneuroim.2010.04.015 20554031

[B27] Silver AC Arjona A Walker WE Fikrig E The circadian clock controls toll-like receptor 9-mediated innate and adaptive immunity Immunity 2012 36 251 261 10.1016/j.immuni.2011.12.017 22342842 PMC3315694

[B28] Feillet CA Albrecht U Challet E “Feeding time” for the brain: a matter of clocks J Physiol Paris 2006 100 252 260 10.1016/j.jphysparis.2007.05.002 17629684

[B29] Carlson DE Are you having a good day: a passing nicety or a fundamental question in the intensive care unit? Crit Care Med 2012 40 344 345 10.1097/CCM.0b013e318232d2e0 22179375

[B30] Chan MC Spieth PM Quinn K Parotto M Zhang H Slutsky AS Circadian rhythms: from basic mechanisms to the intensive care unit Crit Care Med 2012 40 246 253 10.1097/CCM.0b013e31822f0abe 21926587 PMC3977926

[B31] Cao Q Gery S Dashti A Yin D Zhou Y Gu J Koeffler HP A role for the clock gene Per1 in prostate cancer Cancer Res 2001 69 7619 7625 19752089 10.1158/0008-5472.CAN-08-4199 PMC2756309

[B32] Filipski E Levi F Circadian disruption in experimental cancer processes Integr Cancer Ther 2009 8 298 302 10.1177/1534735409352085 20042408

[B33] Bornstein SR Licinio J Tauchnitz R Engelmann L Negrão AB Gold P Chrousos GP Plasma leptin levels Are increased in survivors of acute sepsis: associated loss of diurnal rhythm in cortisol and leptin secretion J Clin Endocrinol Metabol 1998 83 280 283 10.1210/jcem.83.1.4610 9435456

[B34] Carlson DE Chiu WC The absence of circadian cues during recovery from sepsis modifies pituitary-adrenocortical function and impairs survival Shock 2008 29 127 132 110.1097/shk.1090b1013e318142c318145a318142 10.1097/shk.0b013e318142c5a2 17693947

[B35] Gazendam JA Van Dongen HP Grant DA Freedman NS Zwaveling JH Schwab RJ Altered circadian rhythmicity in patients in the ICU Chest 2013 144 483 489 10.1378/chest.12-2405 23471224 PMC3734886

[B36] Lowry SF The evolution of an inflammatory response Surg Infect (Larchmt) 2009 10 419 425 10.1089/sur.2009.018 19792838 PMC2847835

[B37] Lowry SF The stressed host response to infection: the disruptive signals and rhythms of systemic inflammation Surg Clin North Am 2009 89 311 326 vii 10.1016/j.suc.2008.09.004 19281886 PMC2714172

[B38] Cardone L Hirayama J Giordano F Tamaru T Palvimo JJ Sassone-Corsi P Circadian clock control by SUMOylation of BMAL1 Science 2005 309 1390 1394 10.1126/science.1110689 16109848

[B39] Cassone VM Effects of melatonin on vertebrate circadian systems Trends Neurosci 1990 13 457 464 10.1016/0166-2236(90)90099-V 1701579

[B40] Godin PJ Buchman TG Uncoupling of biological oscillators: a complementary hypothesis concerning the pathogenesis of multiple organ dysfunction syndrome Crit Care Med 1996 24 1107 1116 10.1097/00003246-199607000-00008 8674321

[B41] Taguchi T Yano M Kido Y Influence of bright light therapy on postoperative patients: a pilot study Intensive Crit Care Nurs 2007 23 289 297 10.1016/j.iccn.2007.04.004 17692522

[B42] Mundigler G Delle-Karth G Koreny M Zehetgruber M Steindl-Munda P Marktl W Ferti L Siostrzonek P Impaired circadian rhythm of melatonin secretion in sedated critically ill patients with severe sepsis Crit Care Med 2002 30 536 540 10.1097/00003246-200203000-00007 11990911

[B43] Wenham T Pittard A Intensive care unit environment Cont Educ Anaesth Crit Care Pain 2009 9 178 183 10.1093/bjaceaccp/mkp036

[B44] Campbell IT Minors DS Waterhouse JM Are circadian rhythms important in intensive care? Intensive Care Nurs 1986 1 144 150 10.1016/0266-612X(86)90092-1 3635563

[B45] Refinetti R Lissen GC Halberg F Procedures for numerical analysis of circadian rhythms Biol Rhythm Res 2007 38 275 325 10.1080/09291010600903692 23710111 PMC3663600

[B46] Logan RW Sarkar DK Circadian nature of immune function Mol Cell Endocrinol 2012 349 82 90 10.1016/j.mce.2011.06.039 21784128

[B47] O'Callaghan EK Anderson ST Moynagh PN Coogan AN Long-lasting effects of sepsis on circadian rhythms in the mouse PLoS ONE 2012 7 e47087 10.1371/journal.pone.0047087 23071720 PMC3469504

[B48] Gogenur I Bisgaard T Burgdorf S van Someren E Rosenberg J Disturbances in the circadian pattern of activity and sleep after laparoscopic versus open abdominal surgery Surg Endosc 2009 23 1026 1031 10.1007/s00464-008-0112-9 18830755

[B49] Gehlbach BK Chapotot F Leproult R Whitmore H Poston J Pohlman M Miller A Pohlman AS Nedeltcheva A Jacobsen JH Hall JB Van Cauter E Temporal disorganization of circadian rhythmicity and sleep-wake regulation in mechanically ventilated patients receiving continuous intravenous sedation Sleep 2012 35 1105 1114 22851806 10.5665/sleep.1998 PMC3397814

[B50] Farr L Todero C Boen L Reducing disruption of circadian temperature rhythm following surgery Biol Res Nurs 2001 2 257 266 10.1177/109980040100200405 11876465

[B51] Antoch MP Chernov MV Pharmacological modulators of the circadian clock as potential therapeutic drugs Mutat Res 2009 679 17 23 20161366 10.1016/j.mrgentox.2009.07.015 PMC2778065

[B52] Albrecht U Circadian clocks and mood-related behaviors Ann Med 2010 42 241 251 10.3109/07853891003677432 20350255

[B53] McClung CA Circadian rhythms and mood regulation: insights from pre-clinical models Eur Neuropsychopharmacol 2011 21 Suppl 4 S683 S693 21835596 10.1016/j.euroneuro.2011.07.008 PMC3179573

[B54] Hatori M Vollmers C Zarrinpar A DiTacchio L Bushong EA Gill S Leblanc M Chaix A Joens M Fitzpatrick JA Ellisman MH Panda S Time-restricted feeding without reducing caloric intake prevents metabolic diseases in mice fed a high-fat diet Cell Metab 2012 15 848 860 10.1016/j.cmet.2012.04.019 22608008 PMC3491655

[B55] Sprouse J Pharmacological modulation of circadian rhythms: a new drug target in psychotherapeutics Expert Opin Ther Targets 2004 8 25 38 10.1517/14728222.8.1.25 14996616

[B56] Lanfumey L Mongeau R Hamon M Biological rhythms and melatonin in mood disorders and their treatments Pharmacol Ther 2013 138 176 184 10.1016/j.pharmthera.2013.01.005 23348014

[B57] Naus T Burger A Malkoc A Molendijk M Haffmans J Is there a difference in clinical efficacy of bright light therapy for different types of depression? A pilot study J Affect Disord 2013 151 1135 1137 10.1016/j.jad.2013.07.017 23972661

[B58] Hara R Wan K Wakamatsu H Aida R Moriya T Akiyama M Shibata S Restricted feeding entrains liver clock without participation of the suprachiasmatic nucleus Genes Cells 2001 6 269 278 10.1046/j.1365-2443.2001.00419.x 11260270

[B59] Verwey M Amir S Food-entrainable circadian oscillators in the brain Eur J Neurosci 2009 30 1650 1657 10.1111/j.1460-9568.2009.06960.x 19863660

[B60] Froy O The relationship between nutrition and circadian rhythms in mammals Front Neuroendocrinol 2007 28 61 71 10.1016/j.yfrne.2007.03.001 17451793

[B61] Feillet CA Food for thoughts: feeding time and hormonal secretion J Neuroendocrinol 2010 22 620 628 10.1111/j.1365-2826.2010.01998.x 20345747

[B62] Damiola F Le Minh N Preitner N Kornmann B Fleury-Olela F Schibler U Restricted feeding uncouples circadian oscillators in peripheral tissues from the central pacemaker in the suprachiasmatic nucleus Genes Dev 2000 14 2950 2961 10.1101/gad.183500 11114885 PMC317100

[B63] Castillo MR Hochstetler KJ Tavernier RJ Jr Greene DM Bult-Ito A Entrainment of the master circadian clock by scheduled feeding Am J Physiol Regul Integr Comp Physiol 2004 287 R551 R555 10.1152/ajpregu.00247.2004 15155280

[B64] Lamont EW Diaz LR Barry-Shaw J Stewart J Amir S Daily restricted feeding rescues a rhythm of period2 expression in the arrhythmic suprachiasmatic nucleus Neuroscience 2005 132 245 248 10.1016/j.neuroscience.2005.01.029 15802179

[B65] Satoh Y Kawai H Kudo N Kawashima Y Mitsumoto A Time-restricted feeding entrains daily rhythms of energy metabolism in mice Am J Physiol Regul Integr Comp Physiol 2006 290 R1276 R1283 16384858 10.1152/ajpregu.00775.2005

[B66] El-Kadi SW Gazzaneo MC Suryawan A Orellana RA Torrazza RM Srivastava N Kimball SR Nguyen HV Fiorotto ML Davis TA Viscera and muscle protein synthesis in neonatal pigs is increased more by intermittent bolus than continuous feeding Pediatr Res 2013 74 154 162 10.1038/pr.2013.89 23736770 PMC4183190

[B67] El-Kadi SW Suryawan A Gazzaneo MC Srivastava N Orellana RA Nguyen HV Lobley GE Davis TA Anabolic signaling and protein deposition are enhanced by intermittent compared with continuous feeding in skeletal muscle of neonates Am J Physiol Endocrinol Metab 2012 302 E674 E686 10.1152/ajpendo.00516.2011 22215651 PMC3311296

[B68] Gazzaneo MC Suryawan A Orellana RA Torrazza RM El-Kadi SW Wilson FA Kimball SR Srivastava N Nguyen HV Fiorotto ML Davis TA Intermittent bolus feeding has a greater stimulatory effect on protein synthesis in skeletal muscle than continuous feeding in neonatal pigs J Nutr 2011 141 2152 2158 10.3945/jn.111.147520 22013195 PMC3223872

[B69] Yoon JA Han DH Noh JY Kim MH Son GH Kim K Kim CJ Pak YK Cho S Meal time shift disturbs circadian rhythmicity along with metabolic and behavioral alterations in mice PLoS ONE 2012 7 e44053 10.1371/journal.pone.0044053 22952870 PMC3428308

[B70] Luna-Moreno D Aguilar-Roblero R Diaz-Munoz M Restricted feeding entrains rhythms of inflammation-related factors without promoting an acute-phase response Chronobiol Int 2009 26 1409 1429 10.3109/07420520903417003 19916839

[B71] Rossi FM Kringstein AM Spicher A Guicherit OM Blau HM Transcriptional control: rheostat converted to on/off switch Mol Cell 2000 6 723 728 10.1016/S1097-2765(00)00070-8 11030351

[B72] Barclay JL Husse J Bode B Naujokat N Meyer-Kovac J Schmid SM Lehnert H Oster H Circadian desynchrony promotes metabolic disruption in a mouse model of shiftwork PLoS ONE 2012 7 e37150 10.1371/journal.pone.0037150 22629359 PMC3357388

[B73] Li XM Delaunay F Dulong S Claustrat B Zampera S Fujii Y Teboul M Beau J Levi F Cancer inhibition through circadian reprogramming of tumor transcriptome with meal timing Cancer Res 2010 70 3351 3360 10.1158/0008-5472.CAN-09-4235 20395208

[B74] Wu MW Li XM Xian LJ Levi F Effects of meal timing on tumor progression in mice Life Sci 2004 75 1181 1193 10.1016/j.lfs.2004.02.014 15219806

[B75] Redman LM Ravussin E Caloric restriction in humans: impact on physiological, psychological, and behavioral outcomes Antioxid Redox Signal 2011 14 275 287 10.1089/ars.2010.3253 20518700 PMC3014770

[B76] Plunet WT Streijger F Lam CK Lee JH Liu J Tetzlaff W Dietary restriction started after spinal cord injury improves functional recovery Exp Neurol 2008 213 28 35 10.1016/j.expneurol.2008.04.011 18585708

[B77] Hasegawa A Iwasaka H Hagiwara S Asai N Nishida T Noguchi T Alternate day calorie restriction improves systemic inflammation in a mouse model of sepsis induced by cecal ligation and puncture J Surg Res 2012 174 136 141 10.1016/j.jss.2010.11.883 21195419

[B78] Jeong MA Plunet W Streijger F Lee JH Plemel JR Park S Lam CK Liu J Tetzlaff W Intermittent fasting improves functional recovery after rat thoracic contusion spinal cord injury J Neurotrauma 2011 28 479 492 10.1089/neu.2010.1609 21219083 PMC3119327

[B79] Hou C Bolt K Bergman A A general life history theory for effects of caloric restriction on health maintenance BMC Syst Biol 2011 5 78 10.1186/1752-0509-5-78 21595962 PMC3123202

[B80] Froy O Chapnik N Miskin R Relationship between calorie restriction and the biological clock: lessons from long-lived transgenic mice Rejuvenation Res 2008 11 467 471 10.1089/rej.2008.0669 18321196

[B81] Aguilera-Martínez R Ramis-Ortega E Carratalá-Munuera C Fernández-Medina JM Saiz-Vinuesa MD Barrado-Narvión MJ Enteral Feeding via Nasogastric Tube. Effectiveness of continuous versus intermittent administration for greater tolerance in adult patients in Intensive Care JBI Libr Syst Rev 2011 9 S218 S234 27820264

[B82] Campbell IT Morton RP Macdonald IA Judd S Shapiro L Stell PM Comparison of the metabolic effects of continuous postoperative enteral feeding and feeding at night only Am J Clin Nutr 1990 52 1107 1112 2122715 10.1093/ajcn/52.6.1107

[B83] Chen YC Chou SS Lin LH Wu LF The effect of intermittent nasogastric feeding on preventing aspiration pneumonia in ventilated critically ill patients J Nurs Res 2006 14 167 180 10.1097/01.JNR.0000387575.66598.2a 16967399

[B84] Hiebert JM Brown A Anderson RG Halfacre S Rodeheaver GT Edlich RF Comparison of continuous vs intermittent tube feedings in adult burn patients J Parenter Enteral Nutr 1981 5 73 75 10.1177/014860718100500173 6785478

[B85] MacLeod JB Lefton J Houghton D Roland C Doherty J Cohn SM Barquist ES Prospective randomized control trial of intermittent versus continuous gastric feeds for critically ill trauma patients J Trauma 2007 63 57 61 10.1097/01.ta.0000249294.58703.11 17622869

[B86] Marshall A West SH Enteral feeding in the critically ill: Are nurse practices contributing to hypocaloric feeding? Int Crit Care Nursing 2006 22 95 105 10.1016/j.iccn.2005.09.004 16289652

[B87] McClave SA Martindale RG Vanek VW McCarthy M Roberts P Taylor B Ochoa JB Napolitano L Cresci G Guidelines for the Provision and Assessment of Nutrition Support Therapy in the Adult Critically Ill Patient: Society of Critical Care Medicine (SCCM) and American Society for Parenteral and Enteral Nutrition (A.S.P.E.N.) J Parenter Enteral Nutr 2009 33 277 316 10.1177/0148607109335234 19398613

[B88] Steevens EC Lipscomb AF Poole GV Sacks GS Comparison of continuous vs intermittent nasogastric enteral feeding in trauma patients: perceptions and practice Nutr Clin Pract 2002 17 118 122 10.1177/0115426502017002118 16214974

